# Pencil beam scanning proton lattice radiotherapy: single-field versus multi-field optimization

**DOI:** 10.3389/fonc.2025.1716722

**Published:** 2026-01-20

**Authors:** Shouyi Wei, Lee Xu, Hang Qi, Ajay Zheng, Milo Vermeulen, Annemarie Shepherd, Kaled Alektiar, Nancy Y. Lee, Richard Bakst, Chandan Guha, Pingfang Tsai, Minglei Kang, Xiaodong Wu, Irini Yacoub, Jehee Isabelle Choi, Arpit Chhabra, Charles B. Simone, Haibo Lin

**Affiliations:** 1 New York Proton Center, New York, NY, United States; 2 Department of Radiation Oncology, Memorial Sloan Kettering Cancer Center, New York, NY, United States; 3 Department of Radiation Oncology, Icahn School of Medicine at Mount Sinai, New York, NY, United States; 4 Department of Radiation Oncology, Albert Einstein College of Medicine, New York, NY, United States; 5 University of Wisconsin-Madison Department of Human Oncology, Madison, WI, United States; 6 Executive Medical Physics Associates, Miami, FL, United States

**Keywords:** lattice radiation therapy (LRT), MFO, pencil beam scanning, proton therapy, SFO

## Abstract

**Objective:**

To evaluate the advantages and disadvantages of single-field versus multi-field optimization in the clinical implementation of pencil beam scanning (PBS) proton lattice radiotherapy (LRT).

**Methods:**

LRT proton plans were created retrospectively for 12 patients with head-and-neck, thoracic, or abdominal bulky tumors, averaging a gross tumor volume (GTV) of 1011.1 cc (between 333 cc and 3546 cc). The plans were developed in the RayStation treatment planning system (version 2023B), adhering to established consensus guidelines for prescription dose and planning goals. For each plan, 6–8 vertices with an average diameter of 1.4 cm were positioned approximately 3.5 cm apart. The prescription was 18 Gy to each vertex and 3 Gy to the GTV. Single-field optimization (SFO) and multi-field optimization (MFO) techniques were employed. The dosimetric parameters of GTV Dmean, D95%, generalized equivalent uniform dose (gEUD a=-10), vertex D90%, peak-to-valley dose ratio (PVDR), and skin D1% were used for plan quality assessment. Plan robustness was also investigated by comparing dose metrics between the nominal and second worst-case scenarios in the robust analysis.

**Results:**

For all 12 patients, both SFO and MFO plans achieved a PVDR close to 4 across the three treatment sites. No significant differences in primary dose metrics were observed between SFO and MFO plans, except for skin D1%, which was reduced by an average of 25% in the MFO plans (p<0.05). Robustness evaluation indicated larger deviations in PVDR, GTV Dmean, and skin D1% between nominal and second worst-case scenarios for MFO plans compared to SFO (p<0.05).

**Conclusion:**

Both SFO and MFO techniques can be reliably implemented with current proton beam quality standards and advanced treatment planning algorithms. While SFO offers better plan robustness in maintaining the originally optimized metrics under various treatment-related uncertainties, MFO enhances the ability to spare critical organs.

## 1 Introduction

Spatially fractionated radiotherapy (SFRT) delivers highly heterogeneous dose distributions to tumors, creating high-dose regions (peaks) within the tumor while modulating the surrounding tumor microenvironment and preserving vasculature within low-dose regions (1). This unique approach can trigger both bystander and abscopal effects, potentially enhancing the overall tumor response and improving treatment outcomes (2). Initially introduced by Kohler in 1909 (3, 4), SFRT has been used since the 1930s via X-ray beams in the kV range, a technique known as GRID therapy. Modern applications of GRID therapy using megavoltage (MV) X-ray beams have been reported since the 1990s to treat large or recurrent tumors, particularly in patients with prior radiotherapy. Clinical SFRT data have demonstrated benefits such as symptom relief, high response rates, good local control, and minimal to low normal tissue toxicities in the palliative, definitive, and pre-operative settings (5–9). However, conventional 2D GRID therapy has limitations in treating deep-seated tumors due to the increased dose to surrounding normal tissues.

To overcome these limitations, Wu et al. introduced LATTICE Radiotherapy (3D-LRT) (10), an advanced 3D version of SFRT. This technique involves creating multiple spherical high-dose vertices within tumor volumes, strategically placed to achieve high peak-to-valley dose ratios (PVDR)—a critical parameter for effective SFRT—while maintaining prescribed valley dose distributions in non-vertex regions of the gross tumor volume (GTV). This approach minimizes the dose to surrounding normal tissues, reducing associated toxicities.

3D-LRT can be implemented using advanced radiotherapy techniques such as volumetric-modulated arc therapy (VMAT), intensity-modulated radiotherapy (IMRT), and intensity-modulated proton therapy (IMPT), with specific planning protocols detailed in Wu’s work. Several clinical studies (11–18) have shown that LRT can improve local tumor control and reduce toxicities, particularly for large tumors in challenging treatment sites such as the pelvis and lung. Recently, consensus reports (2, 19, 20) have provided guidelines for designing prospective clinical trials for SFRT, addressing patient eligibility, SFRT techniques, dose prescriptions, fractionation schedules, target and normal tissue dose constraints, and clinical endpoints.

Despite growing interest in LRT within the radiation oncology community (21), its potential remains underexplored, particularly considering recent technological advancements. Photon-based LRT, typically implemented using VMAT, has been the primary focus of clinical studies (11–18). However, there is increasing interest in proton-based LRT using IMPT (22–24), driven by the unique physical properties of protons, including a sharp dose fall-off at the Bragg peak that minimizes the exit doses to normal tissues beyond the treatment volume. This feature provides additional dosimetric advantages for SFRT, potentially allowing for dose escalation or a simultaneous integrated boost (SIB), leading to the potential for improved tumor control and response.

Recently, Mossahebi et al. introduced a robust proton lattice planning strategy using two orthogonal proton beams, which was implemented in a pilot clinical study (25). However, literature on IMPT-based proton pencil beam scanning (PBS) planning approaches for SFRT remains limited. Given the importance of plan quality and efficient treatment planning methods (25), a comprehensive evaluation and validation of various planning strategies is necessary to fully explore the potential of proton therapy for LRT. More recently, Setianegara et al. (26) developed and evaluated proton GRID and LATTICE (pSFRT) plans, demonstrating higher PVDRs and improved OAR sparing compared with photon virtual GRID plans.

In this study, we investigated the robustness of proton LRT plans against clinical uncertainties for both single-field and multi-field optimization planning techniques using a commercial treatment planning system (TPS). The treatment planning protocol has been standardized to generate reliable, safe, and effective proton LRT plans suitable for clinical use. Validation of this protocol has strengthened our confidence in the robustness of the plans based on key parameters deemed essential for consistent and reliable SFRT delivery. Notably, these methods have already been implemented in our clinic for patient treatments.

## 2 Methods and materials

### 2.1 Study design

In this retrospective study, 12 patients previously treated with proton therapy across three anatomical sites were replanned using the SFRT technique. The cohort included 3 head-and-neck (H&N) cases, 5 thoracic cases, and 4 abdominal cases, predominantly involving sarcomas. Disease sites, tumor stages, tumor sizes, dose prescriptions, and fractionation schemes were selected based on recently published SFRT clinical trial consensus guidelines (1).

Treatment planning was performed using the RayStation 2023B (RaySearch Laboratories AB, Stockholm, Sweden) treatment planning system, adhering to the photon-based SFRT guidelines established by Wu et al. (10). The planning process incorporates elements such as dose prescription, beam arrangement, vertex diameters, an algorithm for vertex placement and number, organ-at-risk (OAR) constraints, skin dose reduction, and single- or multi-beam contributions to individual vertices.

For each patient, the gross tumor volume (GTV) was contracted by 1.0 cm in superficial tumors or 1.5 cm in deep-seated tumors to create an inward margin of safety. Spherical vertices were placed within the contracted GTV. Treatment plans were optimized using IMPT with a prescribed vertex dose of 18 GyE and a GTV dose of 3 GyE in a single fraction. Dosimetric metrics, including GTV D90%, Dmean, generalized equivalent uniform dose (gEUD, with a = -10), peak-to-valley dose ratio (PVDR) defined as GTV D2%/GTV D50%, and skin D1%, were analyzed against the corresponding planning goals. Additionally, the robustness of the LRT plans was evaluated across 21 perturbation scenarios, accounting for a 3.5% range uncertainty and a 5 mm setup uncertainty for lung and abdominal cases or 3 mm setup uncertainty for head and neck (H&N) cases.

### 2.2 Vertex placement

To facilitate vertex placement, we developed a reference table based on preliminary tests using spherical targets of various sizes (22). This table guides the selection of appropriate vertex diameters and spacing based on target size to achieve a GTV coverage ratio of 1-3%. **Table 1** summarizes the vertex parameters used in the LRT plans, including vertex diameter, spacing, number, volume, and the ratio of vertex volume to GTV for planning purposes. This approach ensures an approximately standard distribution of vertices within the GTV, promoting consistent dose coverage and treatment efficacy.

**Table 1:** Proton Lattice planning geometry metrics based on target volume.

GTV (cc)	Inward margin (cm)	Vertex				V_v_/V_GTV_ (%)
		Diameter (cm)	Spacing (cm)	Number	Volume (cc)	
100-200	1-1.5	1.0	3.0	2-8	1-4	1-2
200-400	1-1.5	1.2	3.5	3-9	2-8	1-2
400-800	1-1.5	1.4	3.5	3-11	4-16	1-2
800-1200	1-1.5	1.6	4.0	6-14	12-30	1.5-2.5
1200-1800	1-1.5	1.8	4.5	6-15	18-45	1.5-2.5
1800-2800	1-1.5	2.0	4.5	9-18	36-84	2-3
>2800	1-1.5	2.2	5.0	>10	>56	2-3

Vertices were manually placed within the GTV following a quasi–regular grid pattern, aiming to maintain approximately uniform separation in both the transverse and axial directions. The center-to-center spacing between vertices was kept within 3–4.5 cm, depending on the prescribed vertex size, which was selected based on the reference table. Whenever feasible, a grid-like arrangement was preserved; however, the final vertex configuration for each case was adapted to the individual tumor geometry, proximity of critical organs at risk, and standard robustness considerations (e.g., setup uncertainties). These case-specific adjustments were made to balance PVDR, target coverage, and organ-at-risk sparing while maintaining a clinically realistic level of plan robustness.

### 2.3 Treatment planning

We propose two planning methods aimed at achieving characteristic LRT dose distribution while minimizing doses to normal tissue. With the first method, each vertex is created by a single vertex-specific field. This approach potentially reduces dose smearing caused by uncertainties from individual fields but may lead to a relatively high entrance dose to normal tissues proximal to the vertices. Careful selection of beam angles is required to avoid irradiating OARs through the entrance path, which can compromise planning efficiency. We avoided the use of noncoplanar fields in this study in order to preserve treatment efficiency, however noncoplanar fields in some cases could offer more flexibility in avoiding OARs and lead to better planning results. To mitigate the limitations of SFO, we also explored a multi-field method, where each vertex is created through multiple vertex-specific fields from different gantry angles with intensity modulation. This approach is anticipated to lower normal tissue and OAR doses at the beam entrance, but the vertex dose may be more sensitive to beam uncertainties.

#### 2.3.1 Single-field optimization method

Between 4 to 6 fields were used for each case, depending on the target location, geometry, and vertex placement. To preserve the sharp lateral penumbra for the vertex fields, the use of a range shifter was minimized. However, a range shifter was employed when needed to ensure uniform coverage of the GTV.

Robust optimization was applied to the GTV to account for 3–5 mm setup uncertainties (3 mm for H&N and 5 mm for thoracic and abdomen) and 3.5% range uncertainty across 14 perturbation scenarios, i.e., setup uncertainties of 3 or 5 mm applied independently in the right–left, superior–inferior, and anterior–posterior directions—or no setup uncertainty in any direction—combined with a 0% or ±3.5% range uncertainty. Robust optimization was not applied to the vertices to maintain their spherical geometry and maximal peak-to-valley dose ratio. SFO was applied to the GTV fields to achieve uniform base dose coverage, while separate vertex fields were used to cover the vertices. Each vertex was irradiated by only a single vertex-specific field, however each field was allowed to cover more than one vertex. Vertices were grouped based on their location using field-specific targets, and attention was given to avoid overlap of entrance dose regions between fields. All fields were included in a single optimization in RayStation, with separate objectives for GTV coverage and vertex boost, so that the two field groups were optimized simultaneously rather than sequentially.

During optimization, ring control structures were employed to improve the dose conformity. These structures consisted of an inner ring (5 mm), a middle ring (10 mm), and an outer ring (30 mm), which were used to enhance the spherical dose conformity of each vertex. Valley control structures were also created to minimize low-dose spills in the valleys by cropping the contracted GTV by 7 mm from the vertices. Spot spacing and weighting were fine-tuned in IMPT to optimize the dose conformity.

#### 2.3.2 Multi-field optimization method

The MFO method used the same field arrangements, structure sets, and robust optimization settings as the SFO method, and separated fields to cover GTV (GTV fields) and vertices (vertex fields) respectively in the treatment plans. Each vertex was irradiated by multiple vertex fields, but not all planned fields. Valley control structures were used to limit the dose contribution from beams passing through the valleys to maximize the PVDR. All GTV fields were optimized simultaneously to achieve uniform coverage of base dose across the remaining GTV and vertices. Dose fall-off objectives were applied to each target vertex to ensure optimal dose conformity. Both vertex and GTV fields were included in one beam set and optimized together to achieve both GTV and vertex coverage, with the dose contribution from each field determined directly by the multi-field optimization.

**Table 2** summarizes the field arrangements used in the MFO plans. Two to four fields were typically used for each vertex, and a range shifter was often avoided when possible.

**Table 2:** Field arrangement and parameters for 12 MFO LRT plans.

Treatment site	Vertex field(s)		GTV fields	
	Number	Range shifter	Number	Range shifter
Lung	2	None	2	2 cm
	3	None	3	2 cm for one beam
	2	None	2	None
	4	None	2	3 cm
	2	None	4	2 cm
Abdomen	3	None	2	2 cm
	5	None	2	3 cm
	4	None	4	2 cm for one beam
	4	None	4	2 cm for one beam
H&N	3	2 cm for one beam	2	5 cm
	1	2 cm for one beam	2	5 cm
	2	2 cm for one beam	2	5 cm

## 3 Results

### 3.1 Planning parameters

**Figure 1:**
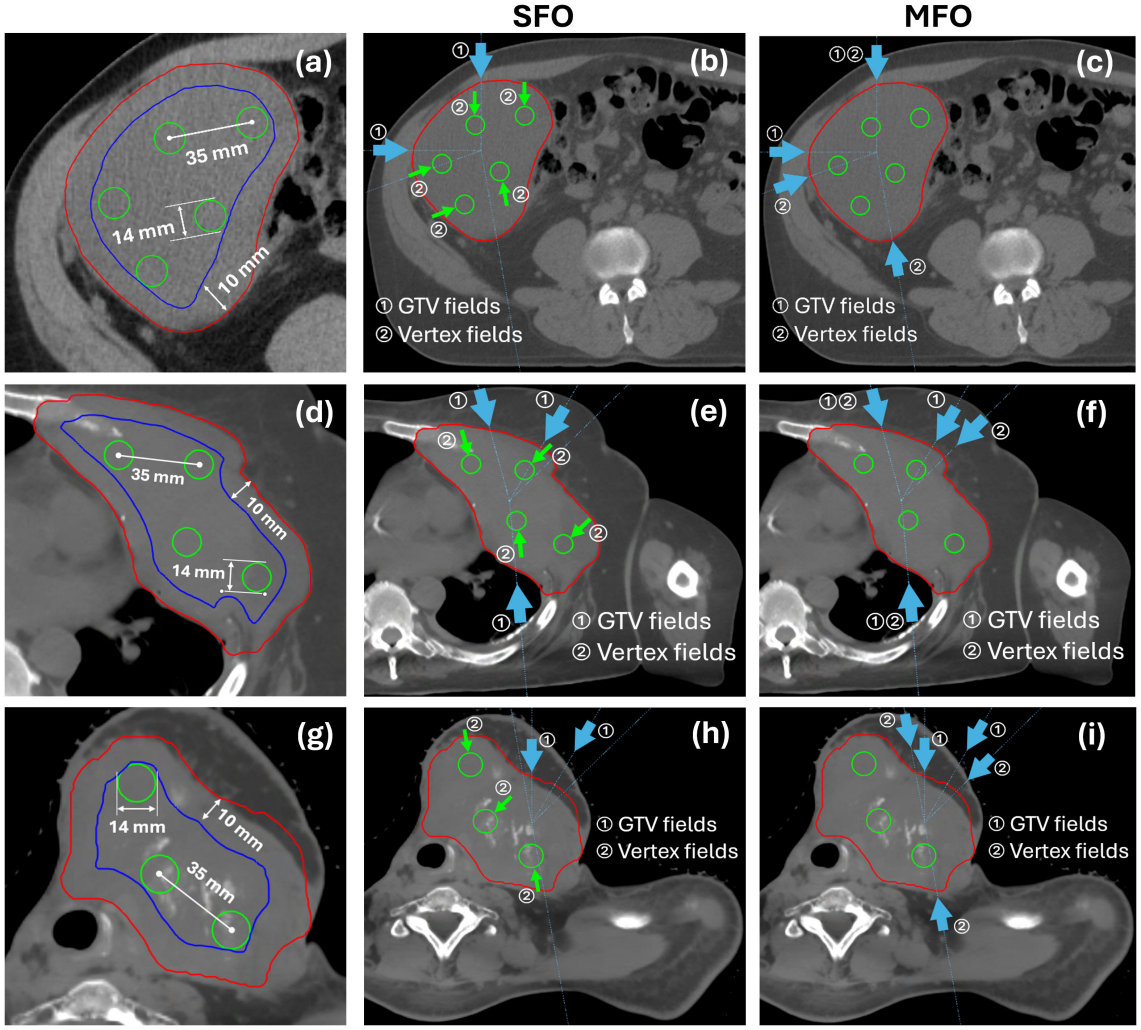
**(a)** Internal GTV and vertex placement in three demonstrative cases for **(a)** abdomen, **(d)** thorax, and **(g)** H&N, respectively. Beam arrangement of GTV and vertex fields for **(b, e, h)** SFO and **(c, f, i)** MFO plans of the same cases.

**Figure 1** illustrates the internal GTV and vertex placement in three demonstrative cases and the corresponding beam arrangement for SFO and MFO plans. **Table 3** summarizes the key planning parameters shared between the single- and multi-field methods. An inward GTV margin of 1cm was applied across all cases. These parameters are consistent with the reference guidelines summarized in **Table 1**.

**Table 3:** Plan parameters of both single-field and multi-field methods.

GTV (cc)	Vertex			V_v_/GTV (%)
	Diameter (cm)	Spacing (cm)	Number	
1011.2 ± 907.7	1.5 ± 0.3	3.7 ± 0.5	3-17	1.6 ± 0.4

### 3.2 Nominal plan quality of H&N plans

Among the three H&N cases, the SFO method resulted in a vertex D90% of 17.8 Gy, an average PVDR of 3.84, an average GTV D95% of 3.1 Gy, a Dmean of 6.3 Gy, and a gEUD (a=-10) of 3.5 Gy. For the MFO method, the vertices D90% was 17.3 Gy, with a comparable average PVDR of 3.85, demonstrating a similar performance to the SFO method. The average GTV D95% increased marginally to 3.2 Gy, while the D50%, D20%, and Dmean were recorded at 4.6 Gy, 8.5 Gy, and 6.2 Gy, respectively. Both SFO and MFO approaches slightly increase GTV D95% above 100% (to approximately 103%–106.7%) as modest over-coverage due to the introduction of high-dose vertices, compared with the uniform plans.

In terms of sparing OARs, the MFO method demonstrated an advantage, particularly for skin. The MFO plan achieved a 35.2% reduction in skin D1% in one of the H&N cases relative to the SFO plan. For the other two cases, we observed 15.0% and 2.1% reductions respectively.

**Figure 2:**
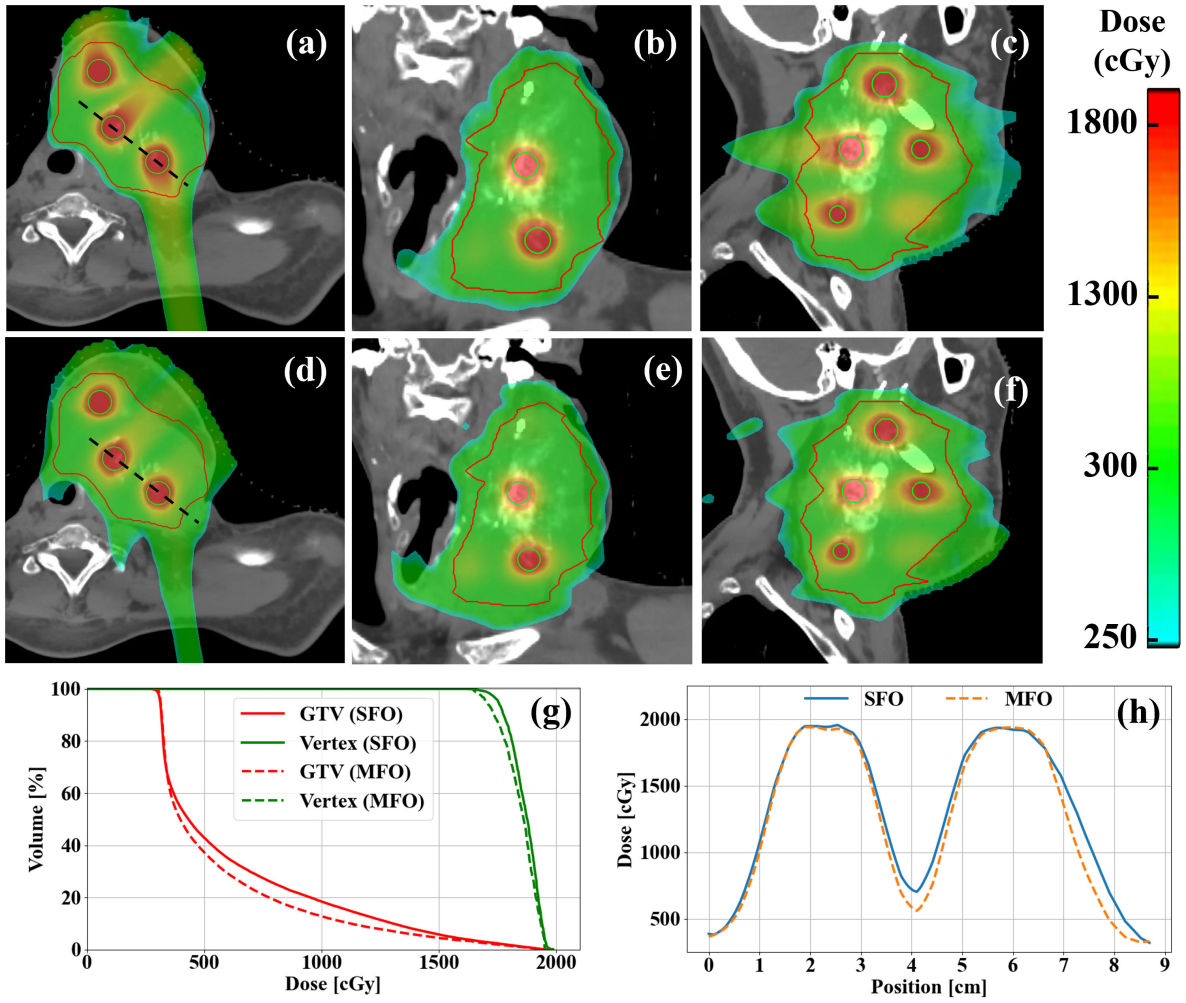
Examples of **(a–c)** SFO and **(d–f)** MFO LRT plans in a H&N case. **(g)** DVH comparisons of SFO and MFO plans. **(h)** Profile comparisons of SFO and MFO in the same case. MFO shows reduced entrance dose compared to the SFO as demonstrated clearly in the dose colorwashes **(a–f)**, while maintaining a similar level of target coverage for both GTV and vertices, as indicated clearly in the DVHs **(g)**, as well as peak-to-valley ratios between vertices.

This improvement is further illustrated in **Figure 2**, where the dose color wash reveals a significant reduction in the entrance dose with MFO, without compromising vertex coverage. The GTV also showed a reduction in the medium-to-high dose volume within the GTV, as confirmed by the dose-volume histograms (DVHs). The PVDR value for both SFO and MFO remains close to 4, underscoring the effectiveness and robustness of LRT treatment planning in both strategies.

### 3.3 Nominal plan quality of thoracic plans

The SFO method resulted in an average PVDR of 4.65 across the five thoracic malignancies cases. For GTV dose metrics, the average D95% was 3.14 Gy, with a Dmean of 5.86 Gy and gEUD (a = -10) of 3.45 Gy. With the MFO method, the average PVDR remained consistent at 4.64. The average GTV D95% was slightly lower at 3.12 Gy, with Dmean of 5.36 Gy and gEUD (a = -10) of 3.5 Gy. The lower Dmean with MFO can be attributed to its ability to optimize the remaining GTV (i.e., GTV minus the vertices) with specific uniformity dose objectives, which was not explicitly incorporated in the SFO method. Additionally, the MFO method significantly reduced skin D1% by an average of 35%.

**Figure 3:**
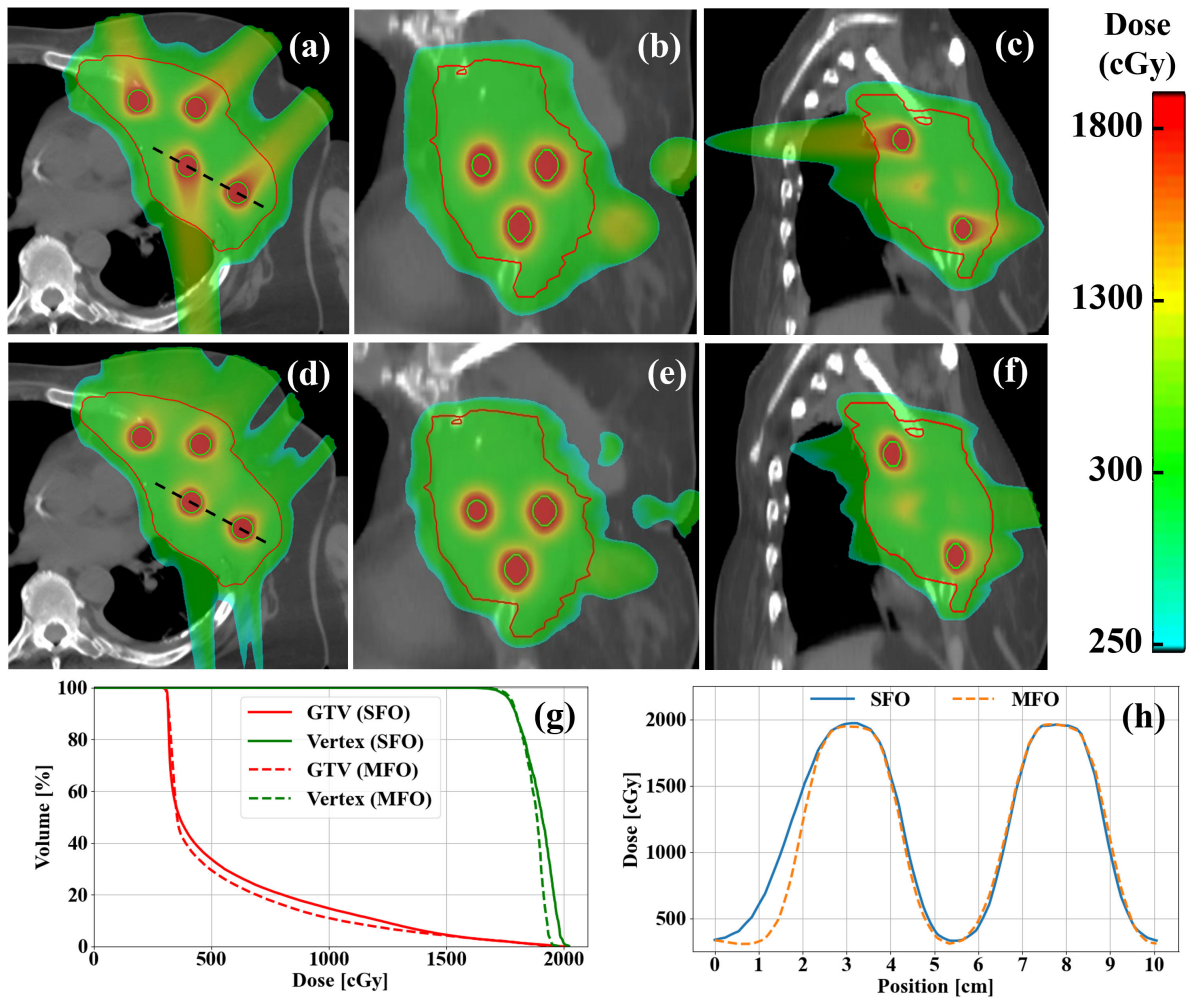
Examples of **(a–c)** SFO and **(d–f)** MFO LRT plans in a thoracic case. **(g)** DVH comparisons of SFO and MFO plans. **(h)** Profile comparisons of SFO and MFO in the same case. MFO shows reduced entrance dose compared to the SFO as demonstrated clearly in the dose colorwashes **(a–f)**, while maintaining a similar level of target coverage for both GTV and vertices, as indicated clearly in the DVHs **(g)**, as well as peak-to-valley ratios between vertices.

**Figure 3** shows a representative thoracic case, where, similar to the H&N case, the entrance dose is greatly reduced in the MFO plan. Vertex dose coverage remains consistent, with minimal changes observed in vertex D90%. Additionally, the MFO plan shows lower volume of medium-to-high dose levels within GTV. The PVDRs for SFO and MFO are nearly identical, further supporting the efficacy of both methods.

The SFO method achieved an average PVDR of 4.68 for the four abdominal cases, with a GTV D95% of 3.1 Gy. The mean dose (Dmean) and generalized equivalent uniform dose (gEUD, a=-10) were 6.2 Gy and 3.1 Gy, respectively. The MFO method yielded superior results, with an average PVDR of 5.03. The GTV D95% was slightly lower at 3.0 Gy, with the Dmean and gEUD (a=-10) being 5.5 Gy and 3.4 Gy, respectively.

In terms of doses to OARs, the MFO method also significantly reduced the skin dose, similar to the H&N and thoracic cases, with the D1% reduced by over 30% in the abdominal cases. Notably, two cases demonstrated a reduction of more than 40%.

### 3.4 Nominal plan quality of abdomen plans

**Figure 4:**
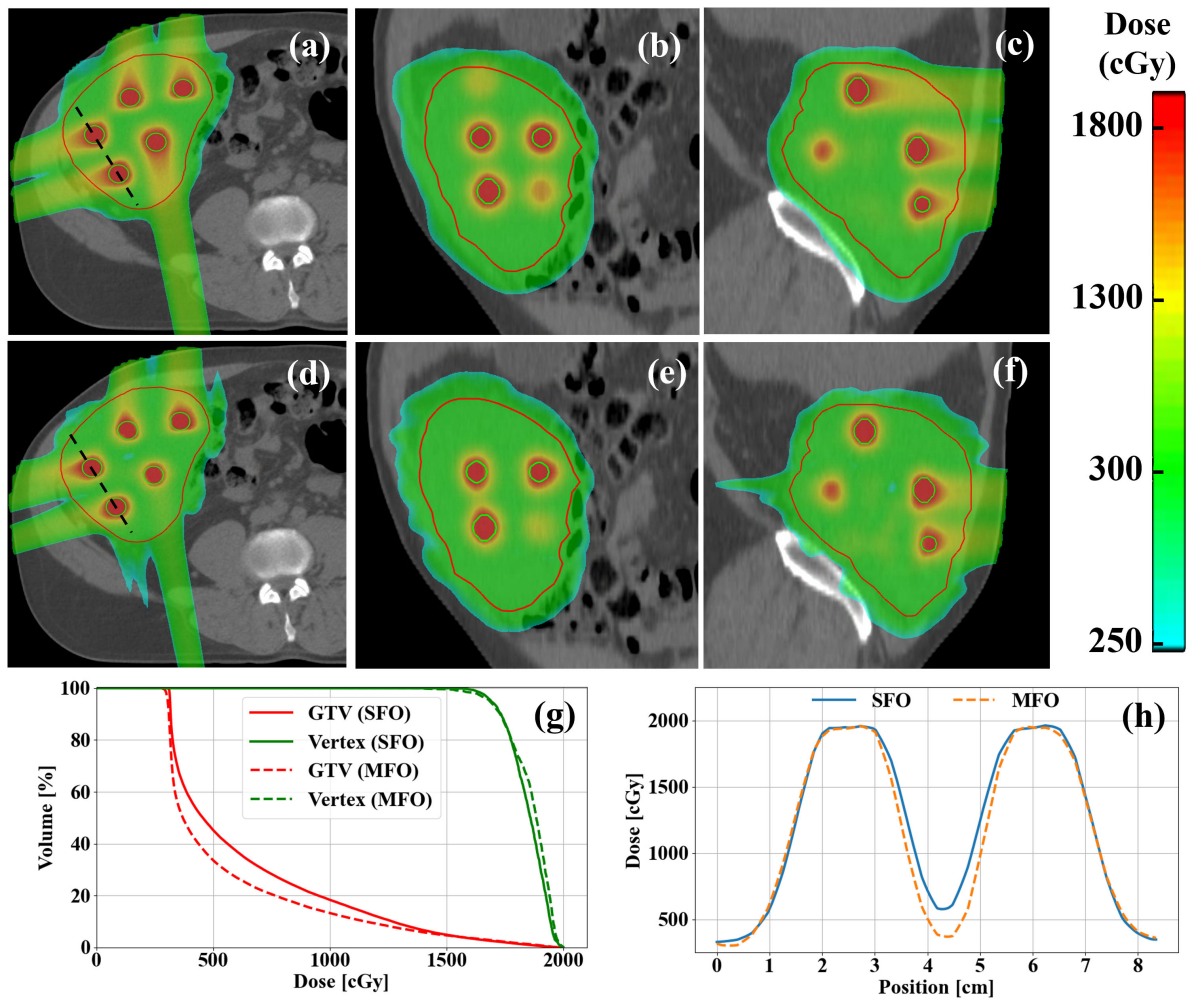
Examples of **(a–c)** SFO and **(d–f)** MFO LRT plans in an abdomen case. **(g)** DVH comparisons of SFO and MFO plans. **(h)** Profile comparisons of SFO and MFO in the same case. MFO shows reduced entrance dose compared to the SFO as demonstrated clearly in the dose colorwashes **(a–f)**, while maintaining a similar level of target coverage for both GTV and vertices, as indicated clearly in the DVHs **(g)**, as well as peak-to-valley ratios between vertices.

**Figure 4** illustrates a representative abdominal case placed with MFO and SFO, showing consistent findings with the H&N and thoracic cases regarding entrance dose reduction, vertex and GTV dose coverage, and PVDR ratios.

A statistically significant reduction in skin D1% was observed between the MFO and SFO plans (p < 0.01), with MFO achieving an average reduction of approximately 25%. Additionally, MFO delivers a GTV Dmean dose much closer to the GTV prescription target, reflecting improved dose uniformity. This improvement is largely attributed to the significantly lower entrance dose in MFO plans, directly correlating with the observed reduction in skin D1%.

**Figure 5:**
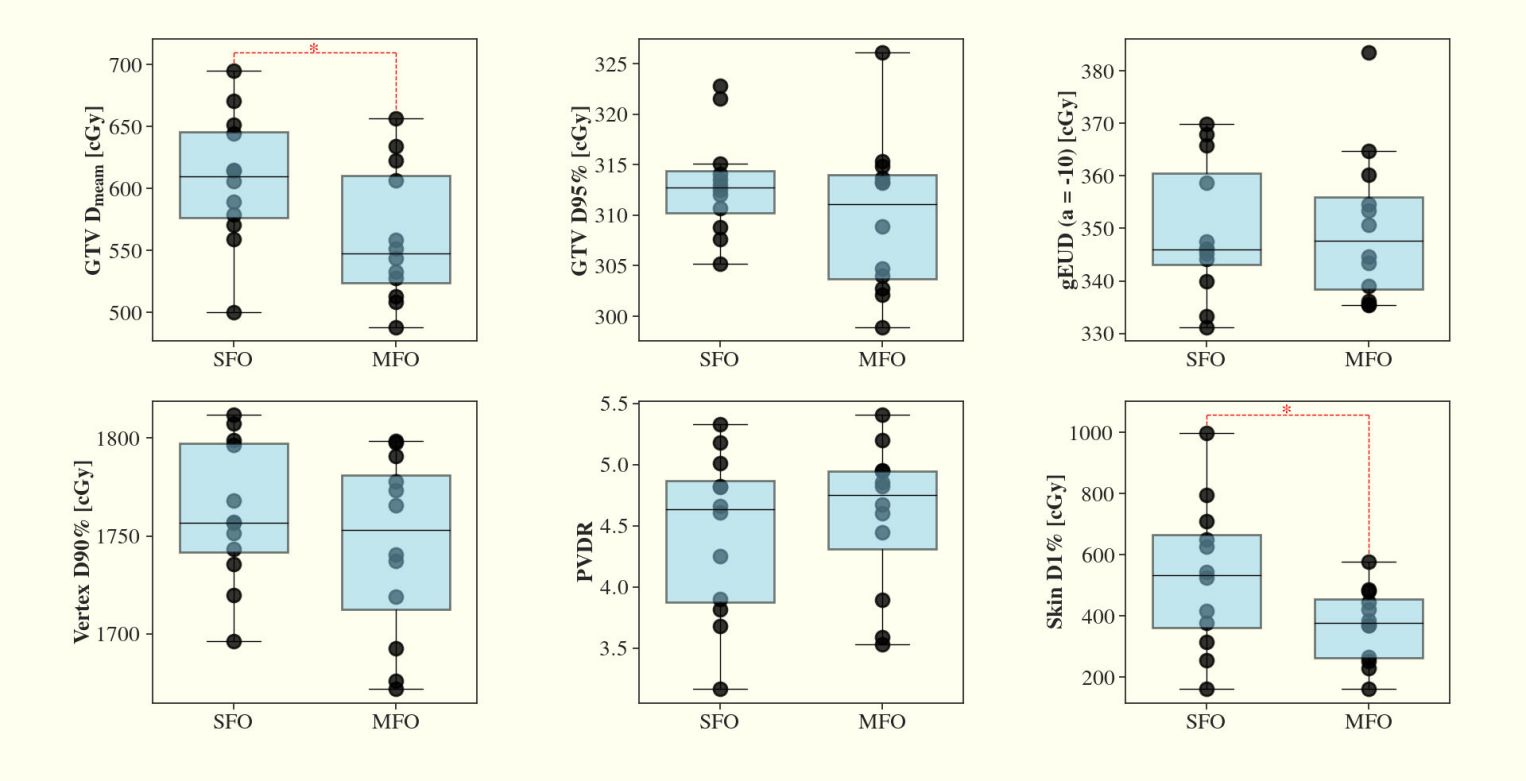
Comparisons of SFO and MFO in LRT dose metrics of GTV Dmean, D95%, gEUD (a=-10), vertex D90%, PVDR and skin D1% across the 12 planning cases. MFO shows closer GTV dose Dmean to the prescribed 3 Gy and less skin dose D1% compared to the SFO (p<0.05), while other parameters including PVDR remain comparable.

Despite these improvements, no statistically significant differences were observed between the SFO and MFO methods for other metrics, including the PVDR, GTV D95%, and gEUD (a=-10). The results are summarized in **Figure 5**.

### 3.5 Robustness analysis

In the 21 perturbation scenarios analyzed, the average PVDR for the second worst-case scenario decreased by ~ 5%, from 4.43 in the nominal cases to 4.21 using the SFO method. Similarly, for the MFO method, the PVDR dropped by ~ 8%, from 4.57 in nominal cases to 4.21 in the second worst-case scenario. The reduction in PVDR between the nominal and second worst-case scenarios was significantly greater in the MFO plans compared to the SFO plans (p < 0.05), indicating a higher sensitivity of MFO to perturbations in this metric.

In the second worst-case scenario, the MFO plans exhibited an approximate 30% reduction in vertex D90%, compared to a ~10% reduction for the SFO plans (*p* < 0.05), indicating inferior robustness in vertex dose coverage for MFO. Since the vertex contours are fixed and relatively small in size, setup or range perturbations can displace the high-dose regions away from the predefined vertex locations, as reflected by the observed deviations in vertex D90%. However, this does not necessarily indicate a degradation of the spatially fractionated dose distribution within the GTV, as evidenced by the preserved peak-to-valley dose ratio (PVDR).

For both SFO and MFO methods, the GTV D95%, Dmean, and gEUD (a=-10) decreased by 2-6% in the second worst-case scenario compared to the nominal cases. However, these reductions were not statistically significant. 

**Figure 6:**
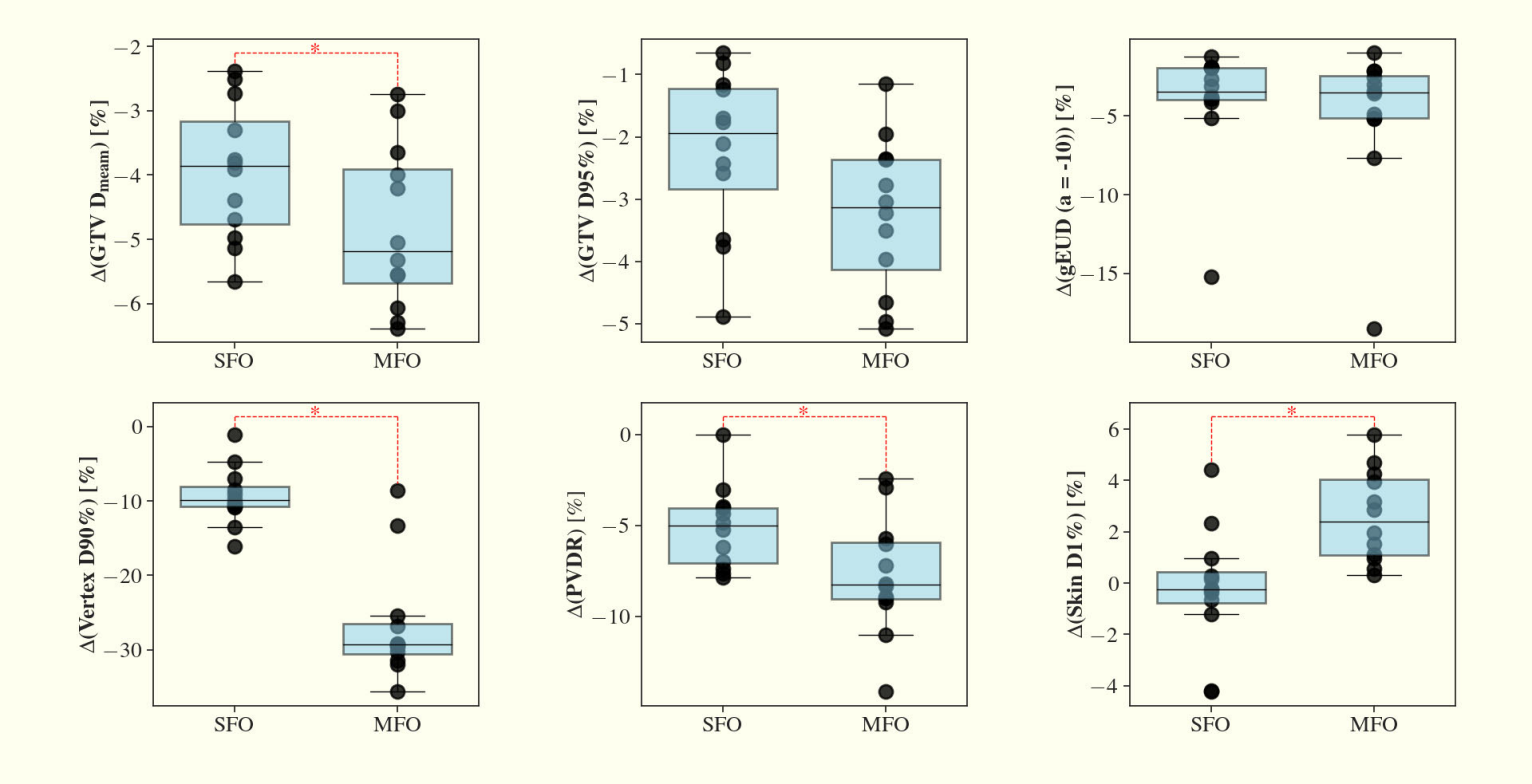
Comparisons of SFO and MFO in the relative changes of second-worst case scenario vs nominal GTV Dmean, D95%, gEUD (a=-10), vertex D90%, PVDR and skin D1% across the 12 planning cases. SFO shows better plan robustness in GTV Dmean, vertex D90%, PVDR and skin D1% (p < 0.05) compared to those of MFO plans.

In terms of skin dose, the second worst-case D1% remained unchanged with 5.3 Gy. In contrast, the increase in D1% was more pronounced for the MFO plans by ~ 2%, rising from 3.70 Gy to 3.80 Gy. Similar to the changes in PVDR, the increase in skin D1% between second worst-case scenarios and nominal was significantly greater in the MFO plans compared to the SFO plans (p < 0.05). These findings are summarized in **Figure 6**.

## 4 Discussion

This study demonstrates and validates the feasibility and efficacy of using proton PBS for 3D lattice radiotherapy (LRT) with both SFO and MFO techniques, which were successfully implemented in our clinic and applied in real patient treatments. Our study not only underscores the high quality of nominal treatment plans, but it also highlights the robustness of these LRT plans under perturbation scenarios. Proton PBS offers highly conformal dose delivery to targeted vertices, achieving excellent peak-to-valley dose ratios, a key metric for effective spatially fractionated radiation therapy. Site-specific studies show that this approach can be adapted for various tumor locations, including those with complex anatomies and in close proximity to different OARs.

As expected, the SFO method results in a higher entrance dose, given that each vertex is irradiated by a single beam. However, it requires less need for robust optimization during the planning process. In contrast, the MFO technique significantly reduces entrance doses but increases the need for robustness considerations during planning. Interestingly, the robustness evaluation for MFO plans shows minimal impact on overall plan quality, particularly for PVDRs, which remained comparable to SFO in nominal and second-worst perturbation scenarios. Although a slightly larger reduction in PVDR was observed with MFO, this decrease was negligible across the 12 patient cases studied, highlighting the value of advanced optimization algorithms in SFRT planning. The observed reduction in skin dose with MFO plans may translate into reduced acute toxicity and improved tolerability. This dosimetric benefit could be especially valuable in dose-escalation settings, where sparing of the skin or chest wall may be critical for treatment feasibility.

To balance entrance dose and plan robustness, 2–3 beams per vertex were utilized in this study for MFO planning. Considering range uncertainties, increasing the number of beams could reduce dose conformity at the vertices in perturbation scenarios and negatively impact PVDRs. One of the remaining challenges in implanting SFRT is the lack of dedicated treatment planning software specifically designed for this technique, which currently hampers planning efficiency. In this study, the arrangements of beams and vertices were largely selected manually, relying on the expert judgment of experienced planners to avoid overdosing on nearby OARs and to manage potential setup-related challenges. As a result, automation of SFRT treatment planning will be essential for improving the workflow efficiency. Such a platform should support full automation of vertex placement, field arrangement, optimization, dose calculation, and extraction of LATTICE-specific metrics. Gaudreault et al. (27) demonstrated automated lattice placement in photon therapy using scripting; however, automation in proton therapy presents added challenges. Beam angle selection must account for anatomical constraints, delivery hardware limitations (e.g., collisions), and clinical considerations such as avoiding beams that traverse sensitive structures or metallic implants. For example, in head and neck cases, posterior oblique beams may traverse healthy lung; and anterior oblique beams may interact with high-density dental fillings, creating uncertainties. The incorporation of artificial intelligence (AI), such as large-scale neural networks (28), could offer automated planning solutions, streamlining the placement of vertices and beams based on clinical indications and optimizing beam configurations. Recent deep learning-based beam selection frameworks (29) have demonstrated promising directions in conventional liver IMPT plans. Integrating AI into TPS would vastly enhance LRT workflow and standardization, making it more suitable for large-scale clinical trials.

Recently, beyond IMPT-based strategies, proton arc-based LATTICE planning approaches have also been explored. Lee et al. (30) demonstrated a proton arc LATTICE technique capable of generating high-quality plans with favorable peak-to-valley dose ratios (PVDR) and gradient indices. Concurrently, Zhu et al. (31) presented a proton arc LATTICE implementation incorporating energy layer and LET optimization, highlighting improvements in dose conformity and organ-at-risk (OAR) sparing. However, since most commercially available proton systems do not support arc delivery and are limited to static field configurations, the present study offers clinically relevant insights for institutions seeking to implement IMPT-based spatially fractionated radiotherapy (SFRT), which is broadly compatible with existing proton delivery platforms. In contrast to the orthogonal-beam strategy proposed by Mossahebi et al. (25), which employs a primary beam and a robustness-compensating beam, the current study adopts a more flexible beam arrangement. This flexibility may offer advantages in cases where beam-path selection is critical for sparing adjacent organs at risk or mitigating setup and anatomical uncertainties. In addition, unlike the approach in (25), we intentionally introduced relatively uniform dose coverage in the valley regions for both SFO and MFO plans, given that adequate valley dose coverage remains an important factor for effective tumor control in SFRT (32).

Currently, no standardized dosimetric metric for SFRT consistently correlates with clinical outcomes, primarily due to the lack of extensive clinical studies. While the equivalent uniform dose (EUD) metric has been used to predict tumor control and normal tissue toxicity in GRID therapy, its application to SFRT is complex and likely needs modifications with sufficient experimental validation (10). The development of new, biologically relevant metrics in the context of SFRT is anticipated as clinical studies continue to advance, aided by the recent publications of consensus guidelines for SFRT trials.

Proton dose delivery, particularly with PBS, is highly sensitive to uncertainties, such as range calibration, beam modeling, anatomical changes, setup errors, and patient motion (33, 34). While the SFO method was relatively stable and less affected by setup uncertainties, other factors such as anatomical changes and organ motion, were not fully assessed in this study. These factors are expected to influence dose conformity at the vertices and PVDRs, though the extent of this impact requires further investigation. In this study, lattice vertex placement followed the methodology proposed by Wu et al. (10), which has been associated with favorable clinical outcomes in photon-based VMAT LATTICE treatments. While the optimality of this design for proton-based LATTICE planning remains uncertain due to the current lack of clinical data, its prior success in photon therapy offers a safe, practical, and standardized starting point. Importantly, this approach does not introduce any apparent risks and enables meaningful cross-modality comparisons between photon and proton implementations. As such, it provides a useful foundation for further development, refinement, and validation of proton-specific LATTICE strategies in future studies.

The PVDR metric used in this work was defined as D2%/D50%, with D50% selected to better represent valley dose characteristics than the alternative Dmean(95–100%), which showed minimal variation across LATTICE plans. In our SFO implementation, higher entrance doses were observed compared to VMAT or MFO, resulting in elevated valley doses along beam paths. PVDRs calculated using the Dp/Dmean(95–100%) definition from Wu et al. (10) yielded higher values than our current method (**Table 4**). Using the D10%/D90% definition from Zhang et al. (1), developed for GRID therapy, SFO plans yielded PVDRs around 4, while MFO plans averaged 3.5 (**Table 4**). This difference arises because D10% values in SFO plans tend to be higher, reflecting greater valley dose heterogeneity due to higher entrance doses. While standardized PVDR definitions facilitate cross-study comparisons, further clinical validation is needed through larger-scale studies employing this planning approach. We also observed that the reported PVDR values were lower than the prescribed value (PVDR = 6). This is likely because the vertex regions typically occupied less than 2% of the GTV, so using GTV D2% as a surrogate for peak dose underestimated the true vertex dose and consequently reduced the reported PVDR values, especially in cases with smaller vertex volumes.

**Table 4:** Plan quality comparison of SFO and MFO LATTICE plans in three tumor size groups.

GTV (cc)	SFO				MFO			
	< 600 cc	600–1000 cc	> 1000 cc	All	< 600 cc	600–1000 cc	> 1000 cc	All
GTV Dmean (cGy)	648.5 (40.8)	577.8 (62.2)	596.9 (36.7)	607.7 (53.4)	618.0 (42.1)	525.0 (26.6)	542.8 (45.1)	561.9 (54.8)
GTV D95% (cGy)	316.1 (7.3)	313.7 (1.3)	309.4 (3.3)	313.1 (5.1)	315.9 (7.3)	311.5 (5.1)	302.1 (2.4)	309.8 (7.7)
GTV gEUD (a = -10)	362.8 (10.3)	345.5 (10.4)	340.6 (6.8)	349.6 (13.0)	365.4 (12.9)	347.2 (6.8)	337.7 (3.8)	350.1 (14.4)
Vertex D90% (cGy)	1741.6 (44.4)	1777.0 (32.1)	1767.3 (30.3)	1761.9 (36.2)	1736.8 (51.5)	1752.6 (52.0)	1745.9 (48.5)	1745.1 (46.3)
PVDR (D2%/D50%)	3.73 (0.45)	4.73 (0.57)	4.85 (0.33)	4.44 (0.67)	3.87 (0.42)	4.81 (0.15)	5.06 (0.32)	4.58 (0.61)
PVDR (Dp/Dmean(95-100%))	5.48 (0.29)	5.74 (0.05)	5.81 (0.12)	5.67 (0.22)	5.44 (0.46)	5.87 (0.12)	6.03 (0.08)	5.78 (0.36)
PVDR (D10%/D90%)	3.98 (0.22)	3.88 (0.29)	4.00 (0.36)	3.96 (0.27)	3.65 (0.18)	3.25 (0.28)	3.49 (0.53)	3.46 (0.37)
Skin D1% (cGy)	428.2 (209.5)	477.1 (224.1)	688.6 (259.4)	531.3 (240.8)	349.2 (129.4)	336.8 (113.8)	426.1 (139.6)	370.7 (122.9)

**Figure 7:**
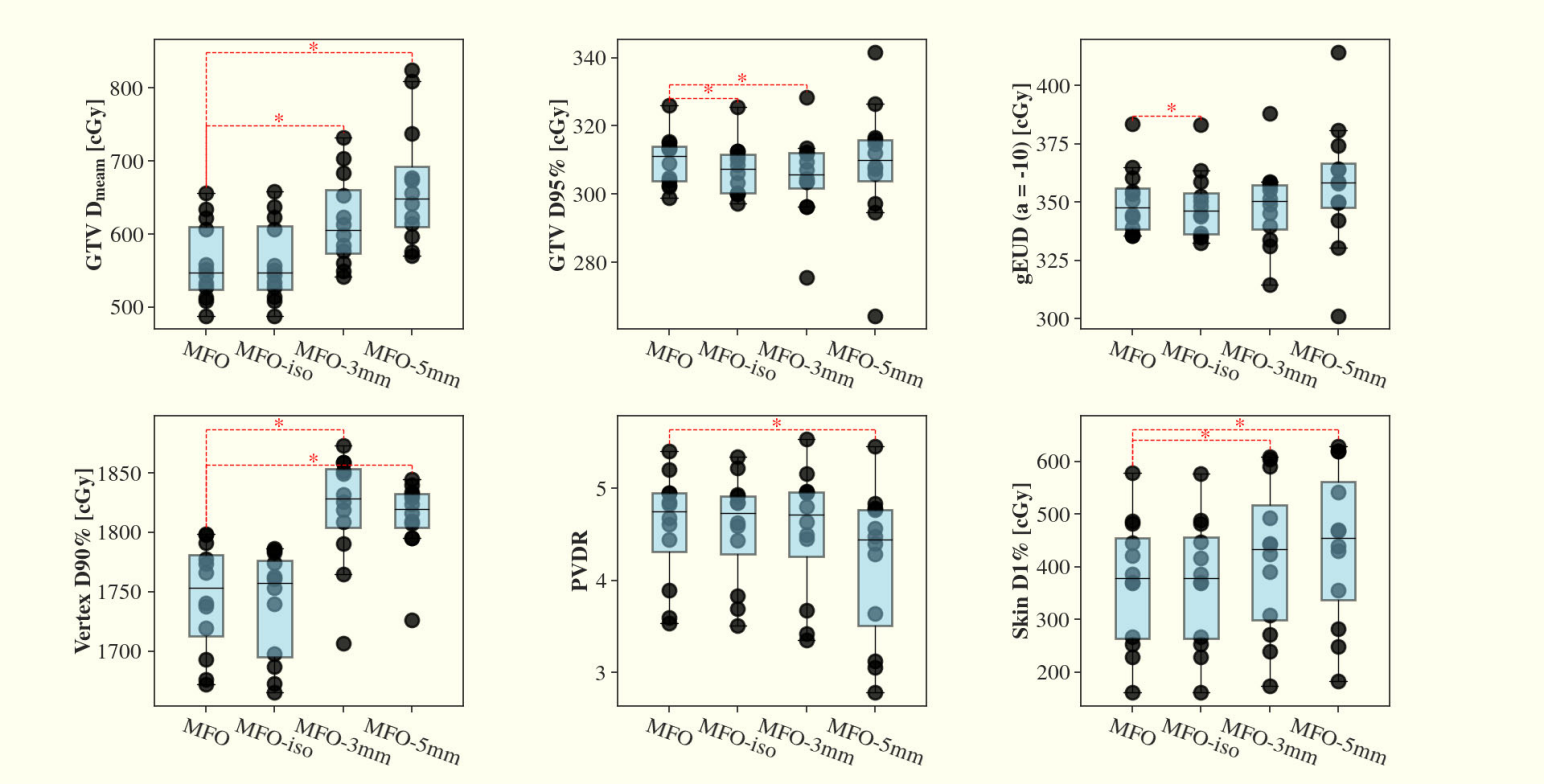
Comparisons of MFO plans, MFO plans under 1 mm isocenter shift between fields (MFO-iso), MFO plans under 3 mm/3.5% and MFO plans (MFO-3mm) under 5mm/3.5% robustness optimization settings (MFO-5mm), in LRT dose metrics of GTV Dmean, D95%, gEUD (a=-10), vertex D90%, PVDR and skin D1% across the 12 planning cases. MFO-iso plans have marginal dosimetric changes compared to original MFO plans. MFO-3mm and MFO-5mm have increased GTV Dmean, vertex D90%, skin D1% compared to original MFO plans (p<0.05). MFO-5mm plans have reduced PVDR (p<0.05) compared to original MFO plans.

**Figure 8:**
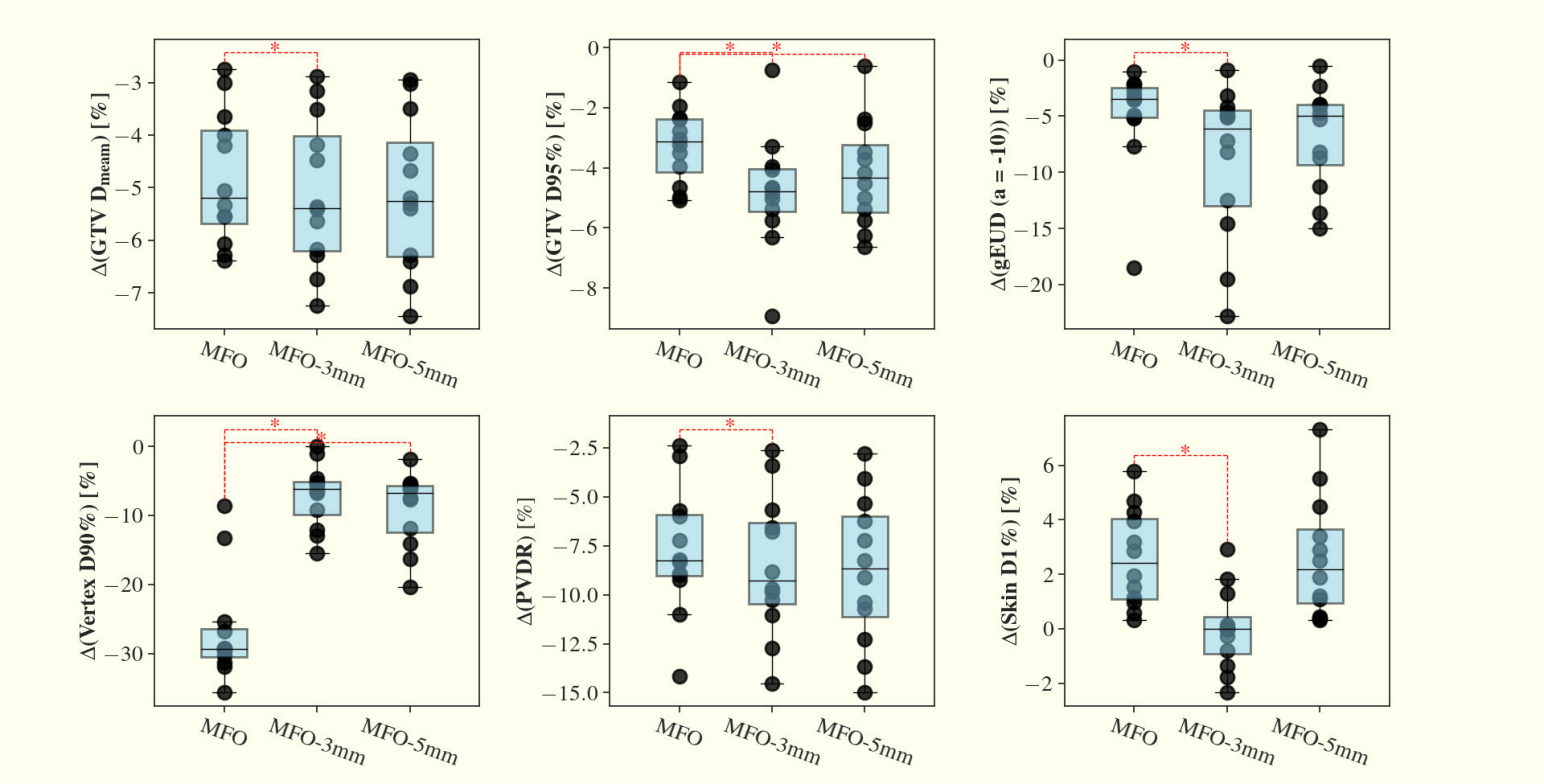
Comparisons of MFO plans, MFO plans under 3 mm/3.5% (MFO-3mm) and MFO plans under 5mm/3.5% (MFO-5mm) robustness optimization settings, in the relative changes of second-worst case scenario vs nominal GTV Dmean, D95%, gEUD (a=-10), vertex D90%, PVDR and skin D1% across the 12 planning cases. Noticeably, there is a significant increase in robustness of vertex D90% of the MFO-3mm and MFO-5mm compared to MFO plans.

**Figure 9:**
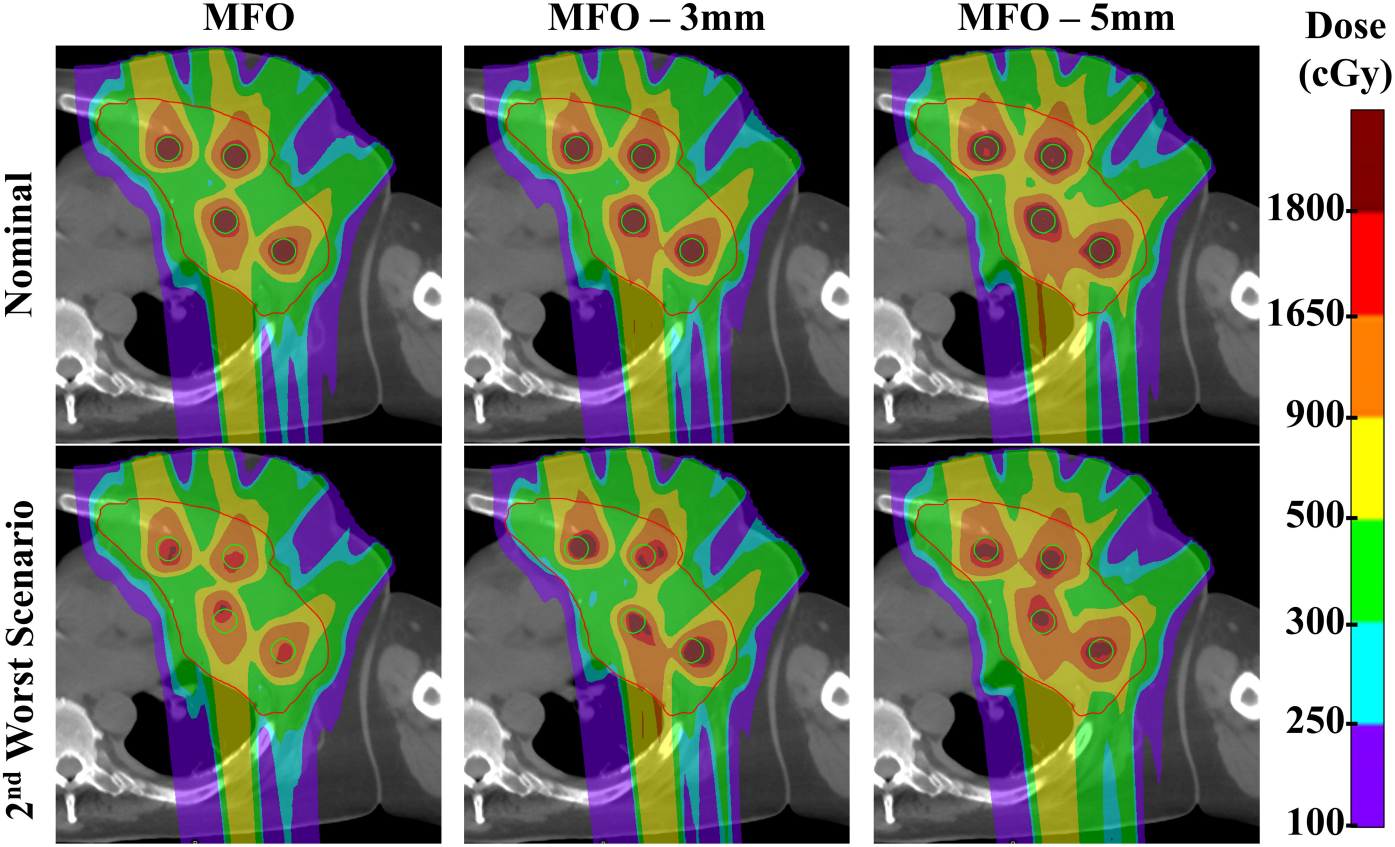
Comparison of dose distribution in the same transverse view of nominal and second-worst scenario MFO plans, MFO plans under 3 mm/3.5% (MFO-3mm) optimization setting and MFO plans under 5mm/3.5% optimization setting (MFO-5mm).

In our current study, robust optimization was not incorporated into the primary SFO or MFO plans. To examine how robustness optimization affects treatment planning outcomes, we applied robust optimization to the MFO approach under two setup uncertainty settings of 3 mm (MFO-3mm) and 5 mm (MFO-5mm), each combined with a 3.5% range uncertainty. We further evaluated the impact of 1 mm inter-field isocenter shifts (MFO-iso). In terms of nominal plan quality (**Figure 7**), MFO-iso plans show only marginal dosimetric differences relative to the original MFO plans. By contrast, MFO-3mm and MFO-5mm plans exhibit increased GTV Dmean, vertex D90%, and skin D1% compared to the original MFO plans (p < 0.05), and MFO-5mm plans demonstrate a reduced PVDR (p < 0.05). In the robustness evaluation (**Figure 8**), the inclusion of robust optimization improved the robustness of vertex D90%, reducing the worst-case decrease from ~30% in the original MFO plans to ~10% in both MFO-3mm and MFO-5mm plans. However, we observed a slight reduction in the robustness of GTV D95%, which may be attributable to increased dose spillage into the low-dose valley regions. Representative dose distributions for the nominal and second-worst-case scenarios for the MFO, MFO-3mm, and MFO-5mm plans are shown in **Figure 9**. As the dose cloud broadens in MFO-3mm and MFO-5mm plans to improve vertex robustness, the valley doses between vertices increase correspondingly.

In our implementation, vertices are confined within the GTV minus a 1-cm margin, so typical setup and range uncertainties are unlikely to displace the high-dose regions outside the GTV. Furthermore, the vertices are separated by sufficient distances such that these uncertainties are unlikely to substantially compromise the PVDR. Importantly, for the high-dose vertices, we do not require the dose peaks to remain at fixed spatial coordinates, provided they remain within the GTV. In this setting, robust optimization tends to enlarge the high-dose “cloud” around each vertex, effectively increasing the vertex size and thereby degrading the PVDR. For both SFO- and MFO-based lattice plans, robust optimization therefore tends to yield suboptimal SFRT characteristics, particularly with respect to peak-to-valley dose ratios.

We also assessed the impact of tumor size on planning outcomes by stratifying the twelve cases into three groups based on gross tumor volume (GTV): 100–600 cc, 600–1000 cc, and >1000 cc. Across all tumor size groups, similar trends were observed between SFO and MFO plans in key dosimetric metrics, including GTV D95%, Dmean, gEUD, vertex D90%, and skin D1% (**Table 4**). However, noticeable differences were observed in PVDR values defined by D2%/D50%, with smaller tumors exhibiting lower PVDRs compared to larger tumors in both SFO and MFO plans. This trend appears to be driven by higher D50% values in the GTV DVHs of smaller targets, suggesting reduced dose uniformity with decreasing tumor size. In contrast, this difference was less pronounced when using the PVDR definition proposed by Wu et al. (10), where Dmean(95–100%) remained more consistent across tumor size groups. We acknowledge that the limited sample size and potential confounding variables, such as tumor location and depth, may reduce the statistical power and generalizability of this analysis. Further studies with larger and more diverse patient cohorts are warranted.

We included free-breathing lung cases exhibiting respiratory motion less than 5 mm, as determined by our clinical motion evaluation software (35). Patients with motion greater than 5 mm were managed with deep-inhalation breath-hold (DIBH) which effectively “freezes” tumor motion with the aid of a commercial respiratory motion management system, thereby further mitigating motion-related uncertainties. In scenarios involving significant organ motion or anatomical shifts not addressed in this study, additional motion management strategies—such as dose repainting—may be necessary to ensure robust and accurate treatment delivery. Future investigations should focus on establishing motion tolerance thresholds, particularly for patients with large tumors or impaired respiratory function, where motion effects could be more pronounced.

## 5 Conclusion

This study successfully demonstrates that proton PBS can provide a superior dosimetric solution for SFRT by leveraging the unique physical characteristics of the proton beams, such as their advantageous exit dose profile. Both SFO and MFO techniques achieve high PVDRs, indicative of high-quality LRT treatment plans. While the SFO technique is less sensitive to setup and range uncertainties, the MFO technique provides superior entrance dose sparing, particularly for skin dose. These findings provide a strong foundation for future clinical trials of LRT using proton PBS, which could pave the way for broader clinical implementation of this advanced radiotherapy technique.

## Data Availability

The original contributions presented in the study are included in the article/supplementary material. Further inquiries can be directed to the corresponding author.
